# Integrative analysis of single-cell transcriptomics reveals age-associated immune landscape of glioblastoma

**DOI:** 10.3389/fimmu.2023.1028775

**Published:** 2023-01-24

**Authors:** Songang Wu, Xuewen Li, Fan Hong, Qiang Chen, Yingying Yu, Shuanghui Guo, Yuanyuan Xie, Naian Xiao, Xuwen Kong, Wei Mo, Zhanxiang Wang, Shaoxuan Chen, Feng Zeng

**Affiliations:** ^1^ Department of Neurosurgery, the First Affiliated Hospital of Xiamen University, College of Chemistry and Chemical Engineering, Xiamen University, Fujian, China; ^2^ Department of Neuroscience, Fujian Key Laboratory of Brain Tumors Diagnosis and Precision Treatment, Xiamen Key Laboratory of Brain Center, The First Affiliated Hospital of Xiamen University, School of Medicine, Xiamen University, Fujian, China; ^3^ National Institute for Data Science in Health and Medicine, School of Life Sciences, Xiamen University, Fujian, China; ^4^ Department of Automation, School of Aerospace Engineering, Xiamen University, Fujian, China

**Keywords:** age, tumor heterogeneity, single-cell RNA sequencing (scRNA-seq), immune microenvironment, GBM, monocyte-derived macrophage (MDM)

## Abstract

Glioblastoma (GBM) is the most malignant tumor in center nervous system. Clinical statistics revealed that senior GBM patients had a worse overall survival (OS) comparing with that of patients in other ages, which is mainly related with tumor microenvironment including tumor-associated immune cells in particular. However, the immune heterogeneity and age-related prognosis in GBM are under studied. Here we developed a machine learning-based method to integrate public large-scale single-cell RNA sequencing (scRNA-seq) datasets to establish a comprehensive atlas of immune cells infiltrating in cross-age GBM. We found that the compositions of the immune cells are remarkably different across ages. Brain-resident microglia constitute the majority of glioblastoma-associated macrophages (GAMs) in patients, whereas dramatic elevation of extracranial monocyte-derived macrophages (MDMs) is observed in GAMs of senior patients, which contributes to the worse prognosis of aged patients. Further analysis suggests that the increased MDMs arisen from excessive recruitment and proliferation of peripheral monocytes not only lead to the T cell function inhibition in GBM, but also stimulate tumor cells proliferation *via* VEGFA secretion. In summary, our work provides new cues for the correlational relationship between the immune microenvironment of GBM and aging, which might be insightful for precise and effective therapeutic interventions for senior GBM patients.

## 1 Introduction

GBM is an aggressive brain cancer with a high incidence rate of 32 per 1,000,000 per year (1). GBM is hard for radical cures surgically and is invalid to radiotherapy and chemotherapy in clinic (2, 3). Patients died by rapid deterioration and shortage of effective medicines. The median survival time of GBM patients is about 15 months after diagnosis (4–6). GBM mainly occurs in the elderly and the median age of first onset is 64 (7). In general, tumors (i.e., leukemia or lung cancer) within younger patients tend to be more malignant (8), whereas GBM patients have worse prognosis with age. However, how and in what extent age influences on the prognosis of GBM are unclear.

Tumor heterogeneity exercises major influence on the prognosis of tumor patients. The heterogeneity in GBM cancer cells has been investigated intensively, which includes but is not limited to the divergence in gene mutations, epigenetic modifications and cell of origins and so on (9–12). The great impact of heterogeneity of tumor microenvironment and immune microenvironment in particular on the tumor progression has been recognized in recent years, which constitutes the theoretical basis of tumor immunotherapy (13–16). The studies on tumor immunology in GBM are limited because of the existence of brain blood barrier (BBB) that blocks the entrance of most of the peripheral immune cells. Bulk omics whose signals summarized over millions of cells are limited to characterize each kind of immune cell populations in GBM tumor environment. Various GBM scRNA-seq data reveal that resident microglia as well as infiltrated peripheral immune cells (including MDMs) contribute to the heterogeneity of GBM immune microenvironment (17–22).

To overcome the batch effects among various scRNA-seq and establish the landscape of immune microenvironment across age, we developed a machine learning method to integrate a variety of scRNA-seq data in public and analyzed the immune cells infiltrating in GBM collected from individuals of various ages. Our data suggested that the heterogeneity of immune cells across age led to the worse OS of aged GBM patients. MDM subpopulation in elderly suppressed the immunologic function and promoted tumor cell growth in a paracrine manner.

## 2 Materials and methods

### 2.1 Integration of single-cell RNA sequencing datasets

We constructed an atlas of cells in GBM by integrating six public scRNA-seq datasets generated by various studies. Because sequencing protocols and experiment conditions are different across these studies, batch effects shall induce an overwhelming number of technical variations in gene expression counts, which impede the integration of different scRNA-seq datasets and the discovery of true biological signals. It is noticed that two kinds of information are immune to technical variations in sequencing and experiment. First, it is the cell-type label that indicates the cell state. Among various GBM datasets, most cell states in tumor environments are similar. Second, the correlation between genes can combat technical variations. Let’s consider the example of face recognition. Even if someone wears a face mask, human visual neurons can precisely recognize the identity of the person. This is because the human brain neural network can utilize the correlation structure among facial features. Similarly, the gene-gene correlation can benefit the detection of true biological signals. The correlation structure among genes can be characterized by relative gene abundance, which is known as gene usage. Based on the above analyses, we designed a factor model called scClassifier to extract gene usages relating to cell states. scClassifier is implemented by using deep neural networks. After training with the cell-type annotated GBM datasets, scClassifier can automatically disentangle the factors regarding cell states and the factors regarding batch effects. The cell state-related factors encode true biological signals, and can be used to predict cell type as well as integrate multiple datasets. Details about scClassifier are given in the following.

Let’s introduce the mathematical notations used throughout the paper first. Let *y* and *z*_1_ denote a factor variable corresponding to cell type and a factor variable accounting for technical variations, i.e., batch effect, respectively. The cell-type annotations *y*_1_,···,*y_N_* of *N* cells are given by users and are discrete variables. Thus, *y* is modeled by the Categorical distribution. Besides batch effect, technical variations of single-cell transcriptomics have multiple resources that have been not well charted. Therefore, *z*_1_ is modeled with the normal distribution. For every cell, the complete hidden cell state *z_y_* is determined by the factors *y* and *z*_1_ with the procedure described as follows,


$$y∼Categorical(\alpha_{0})$$

$$z_{1}∼Normal(\mu_{0},\Sigma_{0})$$

$$z_{y}∼Normal(\mu_{z_{y}},\sum_{z_{y}})$$

where *a*_0_, *μ*_0_, and ∑_0_ are the parameters of prior distributions. In practice, without loss of generality, we choose $a_{0}=\underset{L}{\underset{︸}{\left(1,\cdots ,1\right)}}$ , *L* is the number of cell types. And we choose the mean vector $\mu_{0}=\underset{d}{\underset{︸}{\left(0,\cdots ,0\right)}}$ and the diagonal covariance matrix $\sum_{0}=diag\underset{d}{\underset{︸}{\left(1,\cdots ,1\right)}}$ , *d* is the dimension of the hidden state. In the analyses of the paper, *d* is set as 50. The parameters *μ*_*z*_*y*__ and ∑_*z*_*y*__ are inferred from *y* and *z*_1_ by using a deep neural network.

Once the complete hidden cell state *z_y_* is determined, the generation of single-cell gene expression follows the procedure: first, gene usage *η* is sampled from a Dirichlet distribution, where the parameter is inferred from *z_y_* by using a decoder neural network; second, gene expression counts are generated from *η* by using the multinomial distribution. Specifically,


$$\alpha =Decoder(z_{y})$$

η∼Dirichlet(α)

X∼Multinomial(η)

The parameters of the proposed model are learned by using the stochastic variational inference introduced by the semi-supervised variational autoencoder (23), which can be scalable to large scRNA-seq datasets. To utilize the stochastic variational inference, we introduce the variational distribution,


$$q(X,z_{y},y,z_{1})=q(X)q(z_{y}|X)q(y|z_{y})q(z_{1}|y,z_{y})$$

We use the evidence lower bound (ELBO) as the loss for model training. The deep neural network-based factor model is recently introduced in computer vision. It has been successfully used to factorize various kinds of factors in handwritten digit images (24). In the paper, we extend the deep neural network-based factor model to process single-cell transcriptomics and to extract the variations of gene expression regarding cell states.

### 2.2 Cell type annotation, clustering, and visualization

Besides single-cell integration, scClassifier can be used in a variety of single-cell computational tasks, including the visualization of cell-to-cell variability and cell type annotation. First, the variance distribution model *q*(*z_y_* |*X*) yields 50-dimensional embeddings if gene expression matrix *X* is given. The 50-dimensional embeddings can produce the two-dimensional coordinates of cells through uniform manifold approximation and projection (UMAP). Besides, given gene expression matrix *X*, the model *q*(*z_y_* |*X*)*q*(*y*|*z_y_*) can predict cell type of each cell (cell type annotation). The two-dimensional coordinates and cell type were used to draw the UMAP plots in the paper (25).

To find the subpopulation of every cell type, we needed to perform clustering on the two-dimensional coordinates of cells. Therefore, we built a model that can predict the sub-clusters using a python package Scikit-learn. This model allows for automatic estimation of the number of subpopulations, since the main components of the model are the Dirichlet process.

### 2.3 Cell communication analysis of GBM by iTALK

To explore the growth factor interactions associated with monocytes in GBM, we performed cell communication analysis with an R package iTALK published in 2019 (26). First, we calculated differentially expressed genes (DEGs) between adult and aged patients by using the Wilcoxon signed-rank test. Next, we selected growth factor pairs for our research from the L-R database. Meanwhile, a list of growth factor pairs matching the DEGs was generated by the “FindLR” function in iTALK package. Finally, we sorted the list using the logFC between the adults and aged, and selected monocytes-related growth factor pairs with top absolute value. The growth factor pairs were shown in the Circos plots with the “LRPlot” function. The T cell-associated immune checkpoint interactions within GBM were analyzed with the same procedure.

### 2.4 Differential gene analysis and violin plots

After obtaining the expression matrix corrected for batch effects, we constructed a new Seurat object, and then calculated its differential genes according to the FindMarkers function of the Seurat package. Violin plots are visualized by the VlnPlot function of the Seurat package. Besides, in order to merge different violin plots, we drew the stacked violin plots based on our code.

### 2.5 Data access and survival analysis

For OS data, we used The Cancer Genome Atlas (TCGA) from 606 (Firehose Legacy)) glioblastoma mutiforme samples (GBM_TCGA), and The Surveillance, Epidemiology, and End Results (SEER) Program of the National Cancer Institute (NCI) from 20878 glioblastoma multiforme. TCGA data are accessed by the R package cgdsr (ver 1.3.0). SEER data are downloaded from the SEERStat software (ver 8.4.0.1). To perform survival analysis, the R packages survival (ver 3.3.1) and survminer (ver 0.4.9) are used to calculate and plot the Kaplan-Meier curve. For legacy data, median levels were used to segregate cancer patients according to OS outcome. P value is adjusted with the Benjamini-Hochberg correction method.

### 2.6 Gene ontology analysis

We used the R package clusterProfiler (ver 4.0.5) to perform GO pathway enrichment analysis, where the GO annotation was given in the R package org.Hs.eg.db (ver 3.13.0). The GO enrichment results are shown as the bubble plots with the R package ggplot2 (ver 3.3.6). R version is 4.1.2.

### 2.7 Human tissue specimens and immunohistochemistry

Paraffin-embedded specimens from patients with IV GBM were obtained surgically, from the First Affiliated Hospital of Xiamen University. Collection of all samples was approved by the local ethical committee and the institutional review board of the hospital. Each patient gave written informed consent and patient data have been made anonymous. Detailed information of patients is included in **Supplementary Table 1**. For immunofluorescent staining, blocks were processed at 5 μm. After dewaxing and rehydration, heat mediated antigen retrieval was performed using citrate buffer. And then samples were incubated with lysozyme primary antibody (Abcam, Cat#ab108508) at 1/200 dilution overnight at 4°C. After washing with PBS, the sections were incubated with second antibody conjugated to Cy5 (Jackson ImmunoResearch Laboratories, 1:200) and DAPI (Thermo Fisher Scientific, Cat#D1306) for 1 h at RT. Immunofluorescence images were taken by Leica SP8.

## 3 Result

### 3.1 Senior GBM patients have poor OS

**Figure 1:**
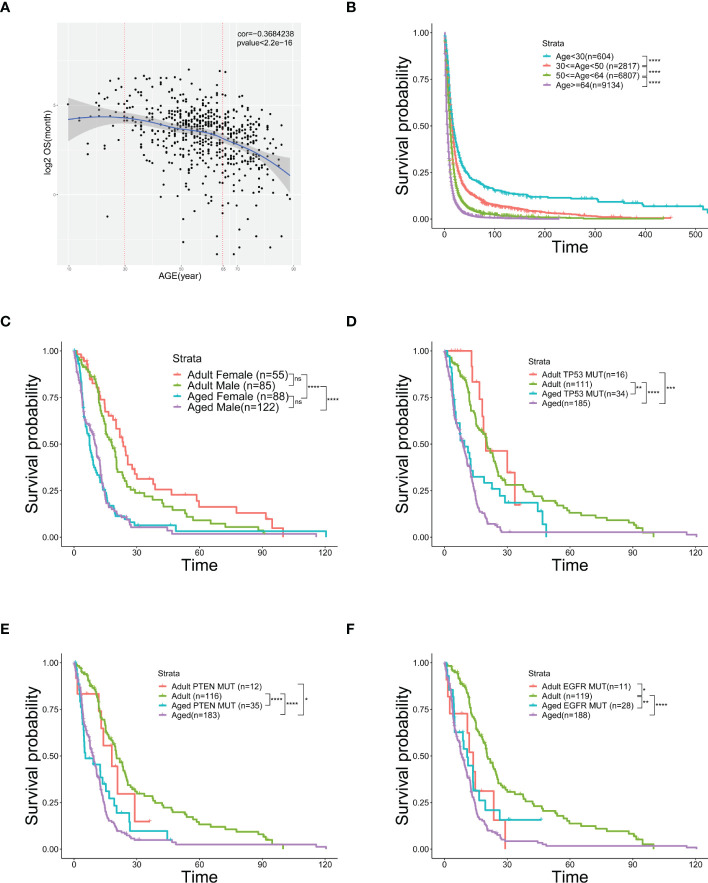
Age is an independent risk factor of prognosis and overall survival. **(A)** Scatter plot of OS (logarithmically transformed) correlated with age from 606 samples in the TCGA database. **(B)** Kaplan-Meier curves of survival analysis used to compare the OS of GBM patients in the SEER database, divided into four groups according to age. **(C–F)** Kaplan-Meier curves of survival analysis used to compare the differences in OS between the two age groups [Adult (30≤age<50) and Aged (age≥64)] with different genders **(C)**, *TP53* mutation **(D)**, *PTEN* mutation **(E)**, or *EGFR* mutation **(F)**. *(P< 0.05), **(P< 0.01), ***(P< 0.001), ****(P< 0.0001), ns (no significance).

Analyzing TCGA and SEER data of GBM patients, we discovered a negative correlation between OS and age (**Figures 1A**, **S1A**) and showed a worse OS in senior GBM patients. Adding with the similar observations from other studies (27), it suggests that age is an independent risk factor of poor prognosis in GBM. Therefore, patients (**Figures 1A**, **S1A**) were grouped by ages and the OS of elder group is significantly poorer than the young group (**Figures 1B**, **S1B**). To clarify that OS was not affected by other factors, we did a survival analysis related to gender and mutation subtype in adults and aged. No significant sexual difference was found in the aged stage though women had a slightly better OS in the adult stage (**Figure 1C**). Neither *TP53* nor *PTEN* mutations alters OS across age (**Figures 1D, E**). Similar results showed that patients with *EGFR* mutation in the aged group still had a poorer OS than those in the adult group, but this mutation could make the adult OS as poor as that in aged group, suggesting that *EGFR* mutations in adults could simulate the changes in old age, and *EGFR* might be involved in the regulation of age-related OS changes (**Figure 1F**). Taken together, age emerges as an independent risk factor of worse OS in GBM.

### 3.2 Integration analysis of single-cell RNA-seq data from adult and aged patients

**Figure 2:**
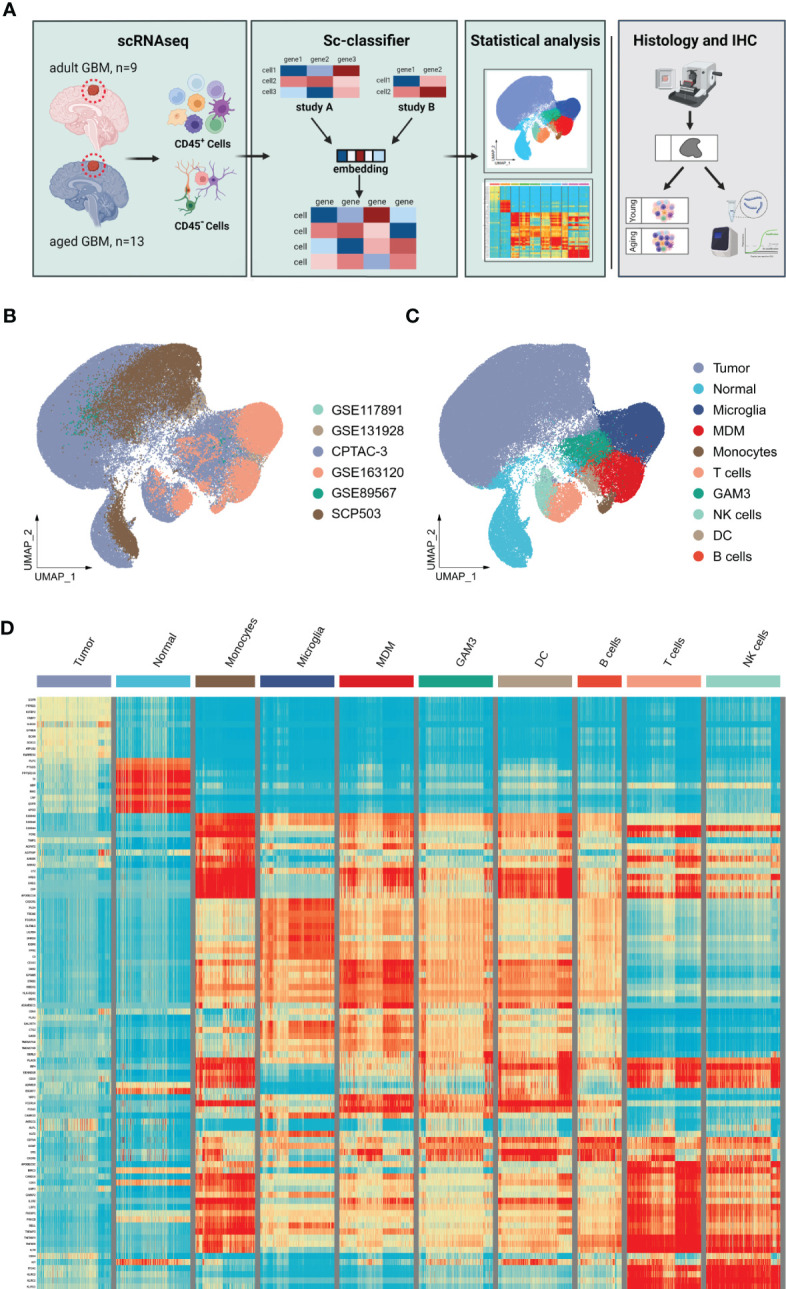
Integration of GBM scRNA-seq containing immune cells and tumor cells. **(A)** Workflow of scRNA-seq integration, data analysis, and validation based on the primary tumors, which are collected from nine adult and thirteen aged patients. **(B)** Corrected UMAP plots colored by the cell types which are identified by marker genes in GBM. **(C)** UMAP plots colored by the scRNA-seq source using our method in each GBM group. **(D)** Heatmap showing relevant expression across different cell subsets. The color is the same as the cell subsets in **Figure 2B**.

The heterogeneity of tumor environment and the heterogeneity of tumor-associated immune cells in particular are unclear in GBM across ages. Herein we focused on immune microenvironment of GBM with scRNA-seq. We analyzed the transcriptome of 23 GBM patients across ages from 6 public scRNA-seq datasets. GBM patients were divided into two groups, adult (20<age< 50, n=10) and aged (age>64, n=13) (**Table 1**). To build a comprehensive immune landscape of primary GBM, 6 datasets were integrated into a huge dataset for analysis (**Figure 2A**). The clustering and UMAP analysis (**Figure S2A, C**) on the huge dataset by traditional Seurat tools were disturbed by ineluctable batch effects that yielded from technical variations in sequencing protocols. Unexpectedly, the biological variations (**Figure S2B, D**) for clustering were removed when batch effects were corrected by classical harmony algorithm. Therefore, we developed a machine learning method named scClassifier to integrate various scRNA-seq datasets to capture the biological variations (Method1). Ultimately, 85,372 cells from the adult tumor samples and 29,634 cells from the aged tumor samples were integrated (**Table 1**, **Figure 2B**). Then tumor cells, normal brain cells, and different kinds of immune cells (**Figure 2C**) were clustered by their universal marker genes (**Figure 2D**).

**Table 1:** Statistical chart shows details of all samples that we collected, such as cell counts and number of patients.

Group	Total Cell Counts	Cell Counts		Number of Patients		Cell Type
		Adult	Aged	Adults	Aged	
1	1901	1645	256	3	1	tumor,immune
2	11786	0	11786	0	6	tumor,immune
3	84969	71053	13916	5	2	tumor,immune
4	15935	12259	3676	1	4	immune
5	415	415	0	1	0	tumor,immune
6	17982	–	–	–	–	tumor
Total	132988	85372	29634	10	13	

### 3.3 More MDMs infiltrate in GBM of aged patients

**Figure 3:**
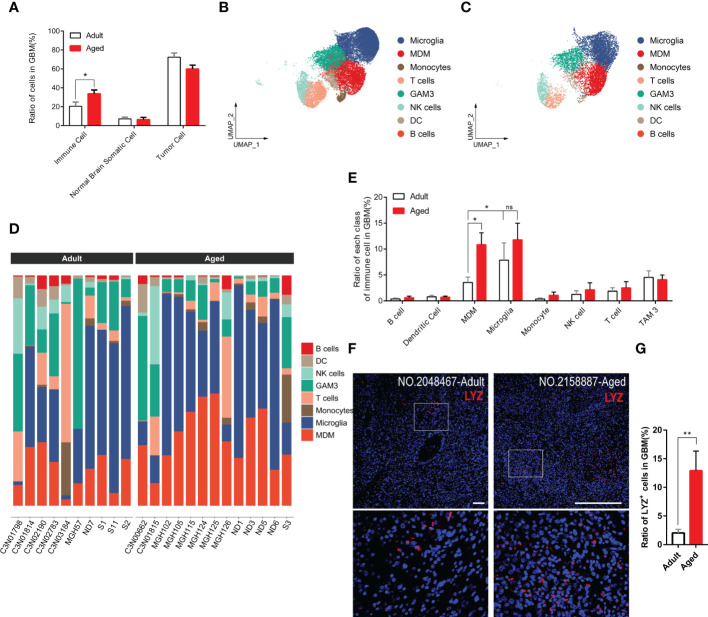
GAM infiltration, especially MDM infiltration, increases more significantly in the aged group. **(A)** Histogram showing the proportion of immune cells, brain somatic cells, or tumor cells in integrated GBM scRNA seq data, grouped by adult and aged (each n=9). **(B, C)** UMAP plots of immune cells in GBM of adult **(B)** and aged **(C)** patients. **(D)** Stacked barplots showing cell types composition of all patients scRNA data from adult group (left) and aged group (right). **(E)** Histogram calculating and comparing all cell types proportion of immune cells in adult group(n=10) and aged group(n=13). **(F, G)** Immunofluorescent staining for lysozyme (MDM marker) in GBM sections from adult group and aged group **(F)**. Amplified areas of white rectangles are shown below and the ratio of *LYZ* ^+^ cells is quantified **(G)**, n=24 fields from 5 GBM samples for each group. All the quantification data are presented as mean ± SEM, two-tailed unpaired Student’s t-test. Scale bars, 100μm. *(P< 0.05), **(P< 0.01), ns (no significance).

Immune infiltration is essential for tumorigenesis (28, 29). Hereby we surveyed the entire infiltrated immune cells in both GBM and normal brain tissues. As expected, the overall immune infiltration was higher in tumors than normal brains (**Figures 3A**, **S3**) (30). Given the top incidence occurs over 65-years-old and 50% of GBM patients are older than 64, the ratio of infiltrated immune cells in aged patients was very close to that in general GBM patients. Compared with aged patients, adult patients had less immune infiltration (**Figure 3A**). 8 subpopulations of immune cells were found in tumor microenvironment. In general, brain-resident microglia composed the majority (31) and the number of other immune cells (e.g., B cells, T cells, DCs) were low no matter in adult or aged patients (**Figures 3B–E**). Comparing each population of immune cells in adult and aged patients, we found the ratio of GAMs was higher in aged patients. GAMs were composed with microglia and MDMs. The MDM rather than microglia population was significantly increased in aged patients (**Figures 3B–E**).

To verify the conclusion above, we stained human GBM tissue samples with *LYZ*, one of the MDM-specific markers (**Figures S4D, S4E**) yielded by feature plot and violin plot. As expected, *LYZ* ^+^ cells were increased in aged GBM patients. (**Figures 3F, G**).

**Figure 4:**
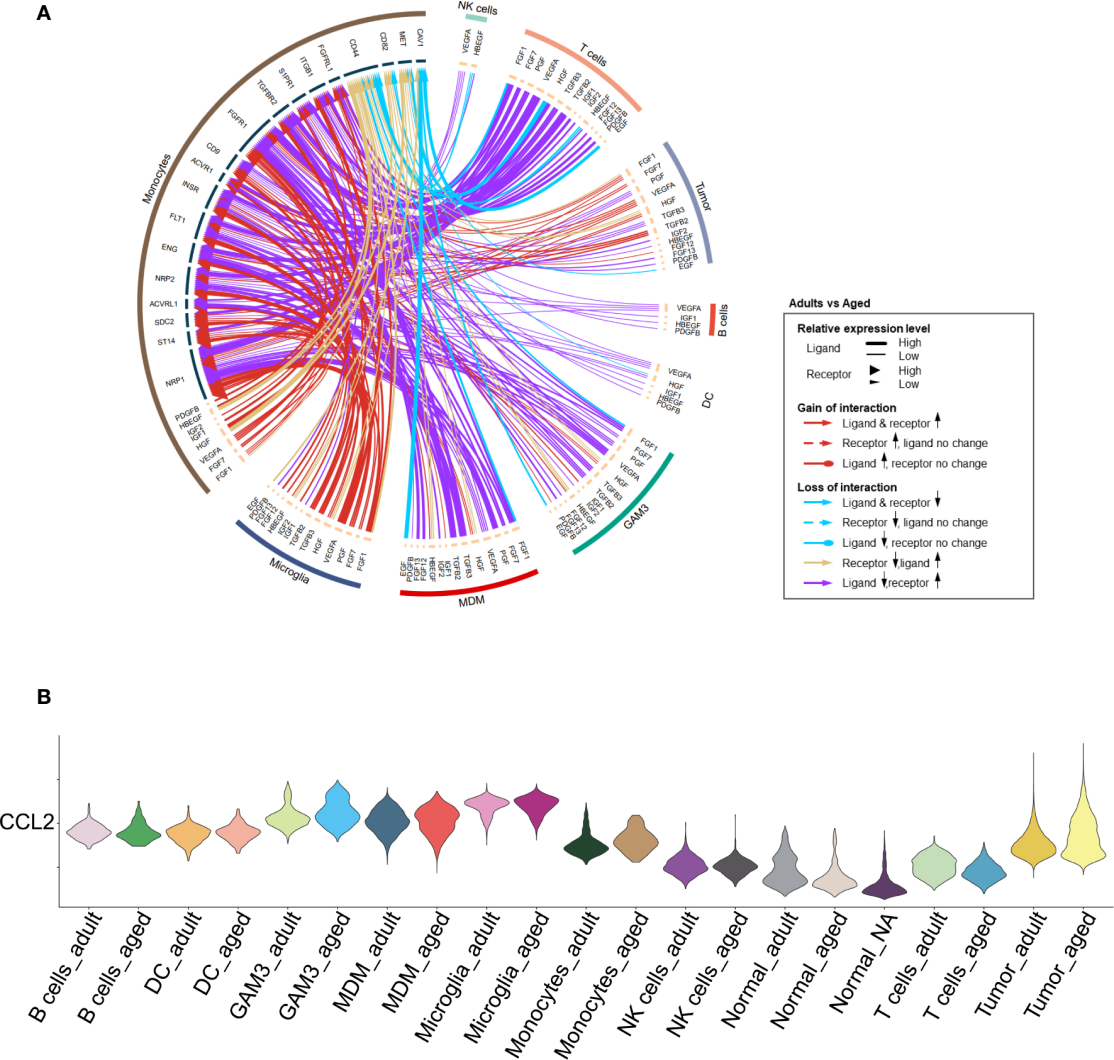
Increased proliferation and recruitment of monocytes. **(A)** Circos plot showing growth factor interactions between monocytes and other cells. The outer ring represents the cell type, and the inner ring represents the gene. The color of the line indicates the direction of upregulation (adult *vs* aged) and the width of the line indicates the degree of upregulation, as shown in the legend. **(B)** Violin plot showing the expression of *CCL2* in each immune cell type, divided by adult and aged cells.

We found that monocyte-associated growth factors were enriched in aged patients which might promote their proliferation (**Figures 4A**, **S5A**). The chemokines for monocytes were evidently up-regulated in tumor cells and other immune cells. For example, *CCL2* was highly expressed in aged tumor cells (**Figures 4B**, **S5B**) to recruit monocyte from peripheral blood (32). Highly expression of *CCL2* is regarded as an unfavorable factor for survival (33). Besides, *CCL2* is also considered to induce M2 polarization of MDMs (34). In all, it may result in enhanced monocytes recruitment from peripheral tissue to GBM and promote them to differentiate into M2 MDMs.

### 3.4 Aged MDMs present features of M2 macrophage

**Figure 5:**
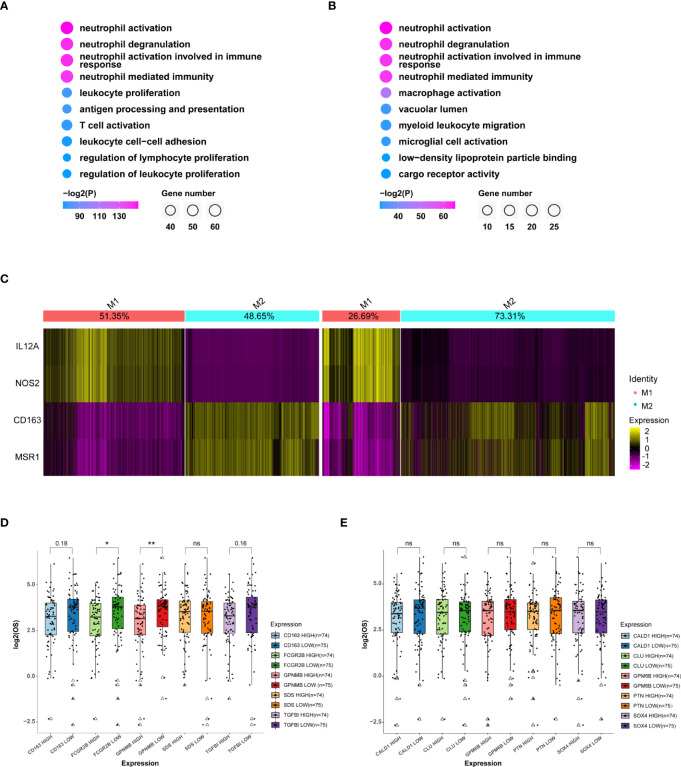
MDMs in the aged group are much anti-inflammatory and unfavorable to OS. **(A, B)** GO (Gene ontology) analysis of top 100 specific genes of MDMs **(A)** or relatively highly expressed genes in aged MDMs **(B)**. Data displaying the top 10 enriched GO terms ranked by p values. The color indicates p values for GO term enrichment and the circle size indicates the number of enriched genes for each GO term. **(C)** Heatmap showing the marker gene signatures of the identified M1 and M2 subtype cells in the adult group(left) and the aged group(right), and the ratio of each subtype in the adult or aged were counted on the top row of columns. **(D, E)** Boxplots showing the differences of OS between high or low expressions of the top 5 genes highly expressed in MDM aged group **(D)** or MDM adult group **(E)**, respectively. Data are obtained from TCGA-GBM database. *P<0.05, **P< 0.01. All the quantification data are presented as mean ± SEM, two-tailed unpaired Student’s t-test. ns (no significance).

To investigate how the heterogeneity of immune cells leads to the worse OS in aged patients, we performed DEG analysis on MDMs and followed by GO analysis for the top 100 genes. Although aged-MDMs rather than adult-MDMs presented typical macrophage signature (**Figures 5A, B**, **S6A**), further analysis using markers of pro-inflammatory features (M1) and anti-inflammatory features (M2) showed that the M2 type was dominant in aged MDMs (**Figure 5C**), which is suppressive to tumor immune activity. In consistent, TOP 5 highly expressed genes in aged-MDMs, i.e., *FCGR2B*, *TGFBI*, *CD163* and *GPNMB* showed negative correlation with the patients’ OS by association analysis of gene expression and patient survival in TCGA (**Figure 5D**). Such correlation was not found in adult MDMs (**Figure 5E**). Thus, the increased immunosuppressive MDMs lead to the poor prognosis and OS in aged patients.

### 3.5 The MDM-B subgroup is significantly increased in older age and is critical for the regulation of age-related OS

**Figure 6:**
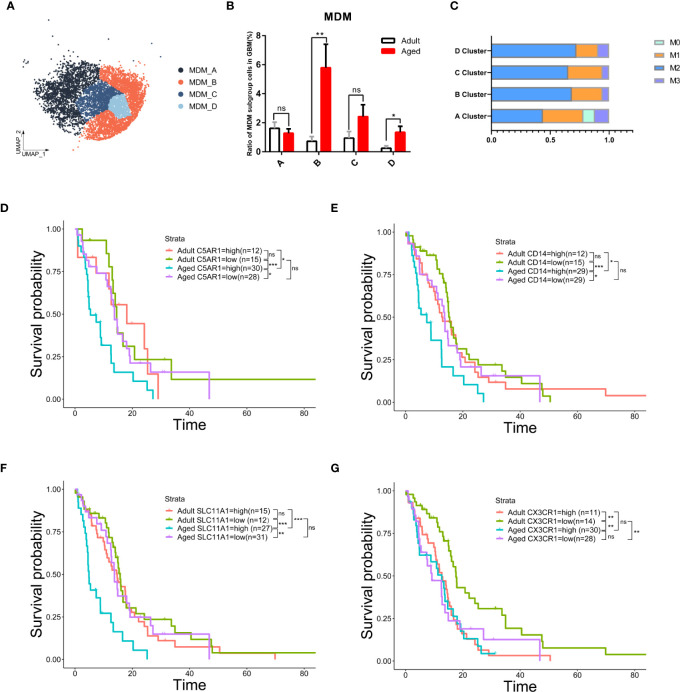
The subgroup of MDM-B but not microglia was the key to age-related OS reduction. **(A)** UMAP plot of four MDM subpopulations in primary GBM. **(B)** Histogram showing the proportion of each MDM subgroup in total GBM cells from the integrated GBM scRNA seq data, grouped by adult (n=10) and age (n=13). ns (no significance). **(C)** Stacked barplot showing macrophage subtype proportion of four MDM subpopulations. M1 and M2 means classical macrophage classification by polarization. M0 means double-negative M1 and M2 features while M3 means double-positive M1 and M2 features. **(D–G)** Kaplan–Meier curves demonstrating the difference of OS in TCGA GBM patients in adult and aged groups with high or low expression of *C5AR1* **(D)**, *CD14* **(E)**, *SLC11A1* **(F)**, or *CX3CR1* **(G)**. All the quantification data are presented as mean ± SEM, two-tailed unpaired Student’s t-test. *(P< 0.05), **(P< 0.01), ***(P< 0.001), ns (no significance).

We further characterized the MDM sub-population that responded to the change of MDMs in both quantity and quality with age. MDMs were divided into four sub-clusters as MDM A\B\C\D (**Figure 6A**). MDM-A was dominant in adult MDMs and decreased with age; whereas the MDM-B increased significantly with age and prevailed in most of aged MDMs (**Figure 6B**). In addition, MDM-B presents typical M2 features (**Figures 6C**, **S7A**). Genes highly enriched in MDM-B (**Figures S7B–D**) and unfavorable to OS were the key factor for poor OS of aged patients. Therefore, a small portion of aged patients with low expression of those genes, such as *C5AR1*, *CD14* and *SLC11A1* had very close OS to that of adult patients (**Figures 6D–F**).

Considering microglia are the most abundant population in all GBM-associated immune cells, microglia from patients across age were further clustered and showed a close proportion of M1/M2 subtypes, excluding the major role of microglia in worse prognosis with age (**Figures 6G**, **S7E, F**). These data suggest the anti-inflammatory MDMs in aged GBM caused the poor OS with age.

### 3.6 Aged MDMs compromise tumor immune response and promote tumor growth in paracrine manner

**Figure 7:**
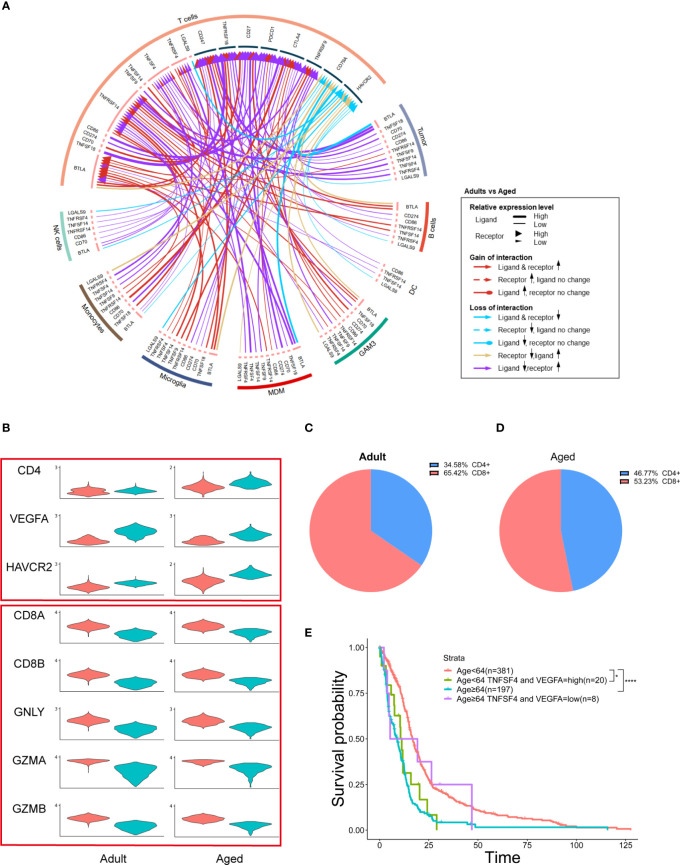
MDMs regulate age-related OS by affecting T cells and secreting growth factors. **(A)** Circos plot showing immune checkpoint interactions between T cells and other cells. The outer ring represents the cell type and the inner ring represents the gene. The color of the line indicates the direction of upregulation (adult *vs* aged) and the width of the line indicates the degree of upregulation, as shown in the legend. **(B)** Violin plot showing the expression of each T cell marker in adult or aged T cells, which divided them into two groups by *CD4* and *CD8* expression. **(C, D)** Pie plots exhibiting the proportion of *CD4* ^+^ and *CD8* ^+^ T cells in adult or aged T cells. **(E)** Kaplan–Meier curves demonstrating the difference of OS in TCGA GBM patients in Age<64 and Age≥64 two groups with high or low expression of *TNFSF4* and *VEGFA*. All the quantification data are presented as mean ± SEM, two-tailed unpaired Student’s t-test. *(P< 0.05), ****(P< 0.0001).

M2 macrophage inhibits T cell function (35) and M2 tumor-associated macrophage restricts T cells from killing tumor cells (36). To examine the immune activity of T cell in GBM, we performed checkpoint communication analysis in both adult and aged patients. We found the immune checkpoint genes were upregulated in T cells from aged patients (**Figures 7A**, **S8**), which suggested an immunosuppressive state in aged GBM. In consistent, cytotoxic *CD8* ^+^ T cells decreased and exhausted *CD4* ^+^ T cells (*HAVCR2* ^+^) increased in aged GBM (**Figures 7B–D**).

MDMs promote tumor growth by secreting growth factors (37). Among all the growth factors detected in GBM, *VEGFA* was highly expressed in MDMs and tumor cells (**Figure S5**), suggesting that increased MDMs could lead to upregulation of *VEGFA* and promote tumor growth.

TCGA data showed that aged patients with reduced *VEGFA* and *TNFSF4* (an immune checkpoint gene) expression had a better OS, which suggests two oncogenic functions by MDMs were synergistic (**Figure 7E**).

## 4 Discussion

**Figure 8:**
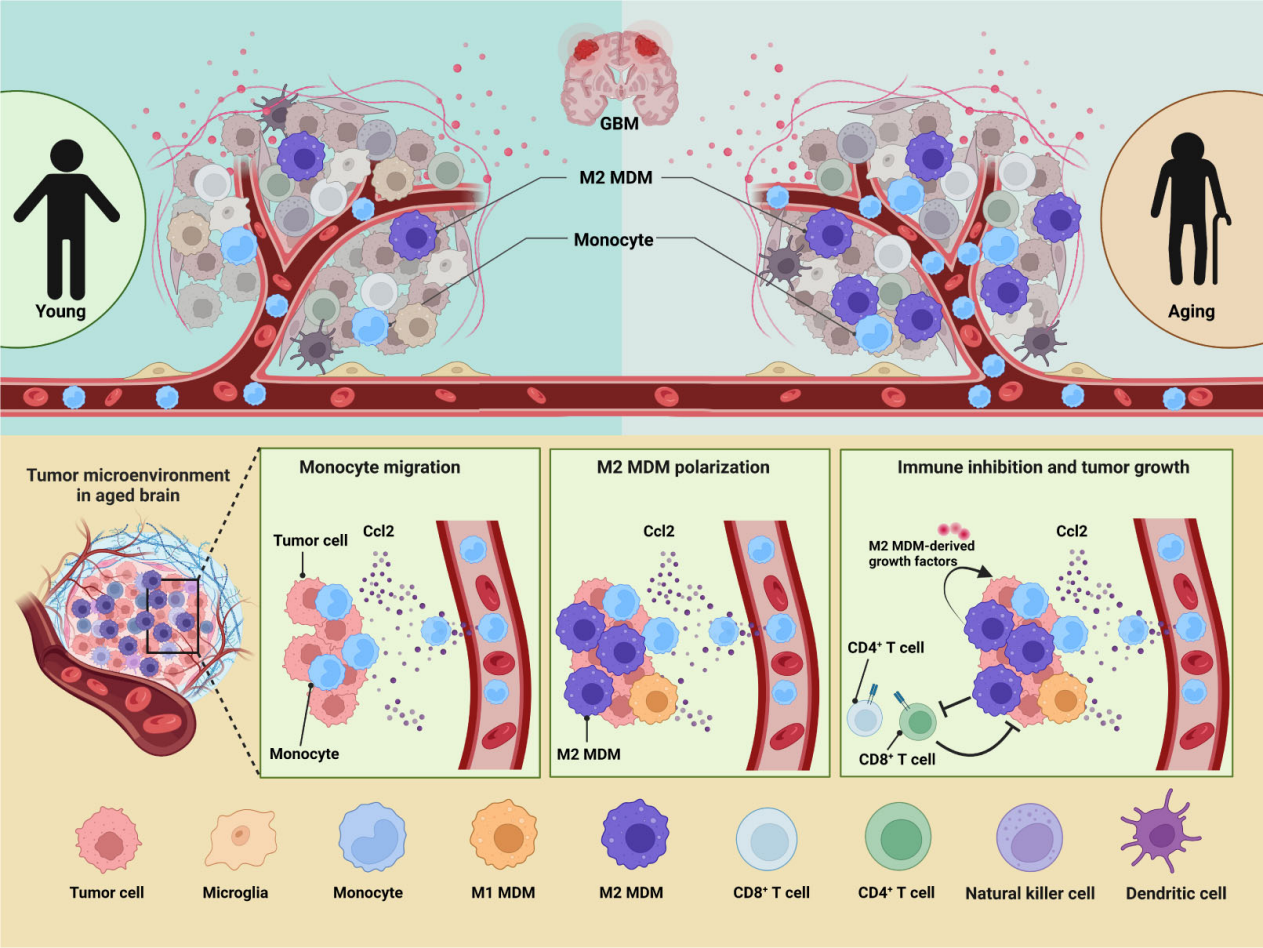
GBM immune heterogeneity across ages. **(A)** A model figure of this article showing how aging influences immune microenvironment of GBM patients.

In this article, we created an atlas of immune cells infiltrating in GBM by holistically analyzing six public scRNA-seq datasets from patients of variant ages. To integrate the six datasets, we developed a machine learning method named scClassifier. Besides, scClassifier shows better performance on cell-type annotation and integration than the standard methods (**Figure S9**). Our data suggested that immune heterogeneity by ages contributed to the worse OS in aged GBM patients (**Figure 8**). The number of infiltrated immune cells was increased with ages and GAMs had the most dramatic change. GAM infiltration has been associated with poor prognosis and OS (38, 39), but the effect of MDMs and microglia may be different by age. Previous studies have not found that MDMs is the immune cell population that changes the most significantly with age. According to TCGA data analysis, our research discovered targeting MDMs is probably more beneficial to improve OS of aged patients but not adult patients.

This difference in the infiltration of MDMs with age may be useful for classification as subtype of GBM. Our study has successfully repeated the used MDM specific markers. Furthermore, we find *FCGR2B*, *GPNMB* etc are probable new MDM specific markers in GBM. These genes can be used to detect the level of MDMs, and to evaluate and predict prognosis and OS of non IDH-mut or EGFR mutation GBM.

MDMs are derived from monocytes. Monocytes are myeloid derived non-resident cells, which need recruitment from extracerebral circulatory vessels. Normal brain tissue is lowly infiltrated by monocytes/MDMs, while in GBM they infiltrate highly. This phenomenon is observed in this article, too. But the difference is that we find a significant increase of MDMs in aged patients. It is previously reported that *CCL2* is secreted abundantly by GBM cells (35, 40). Zhu et al. has found that gliomas cell lines GPL261 and U87 can secret abundant *CCL2 in vitro* (41). Our research shows a high expression of *CCL2* in tumor cells and MDMs, and the expression is much higher in aged group. This may induce a total up-regulation of *CCL2*, and finally induce a hyper accumulation of MDMs, as well as M2 differentiation. Current therapy targeting *CCL2*-*CCR2* axis proves it is a promising target of GBM (42). Whether targeting *CCL2*-*CCR2* axis is only favorable of age-related OS or highly MDM-infiltrated patients needs further exploration.

As for downstream of MDMs, current reports show that MDMs can exert immunosuppressive function through the regulation of T cells. Li et al. has discovered co-culture of naive T cell with glioma-associated MDMs can induce a differentiation into *TGFBI* and *IL-10* secreted *CD4* ^+^ Treg cell (43). Meanwhile, through highly expressing *PD-L1* and *PD-L2*,(*PD-1* ligands) *CD80* and *CD86*(*CTLA-4* ligands) and other immune checkpoint genes, MDMs interact with *CD8* ^+^ T cells and then suppress their cytotoxic function (44). Our research reveals *CTLA-4* and its ligand *CD86* are much more highly expressed in aged T cells and MDMs. This verifies aged MDMs are probably more suppressive for T cells. In addition, many other checkpoint genes are found up-regulated in aged T cells. Among them *TNFSF4* is likely to be a new therapy target to de-repress the negative regulation of T cells from MDMs. After that, *TGFBI* secreted by MDMs can also target *CD8* ^+^ T cells directly, inhibiting its killing effect on tumor cells (45). This is corresponded with the largely upregulated immune checkpoint genes and high expression of *TGFBI* in aged MDMs in our study. Therefore, detecting the expression of these genes for targeted therapy may be able to better treat of GBM.

## Data availability statement

Publicly available datasets were analyzed in this study. This data can be found here: https://www.ncbi.nlm.nih.gov/geo/ Gene Expression Omnibus via GSE numbers: GSE117891 (46), GSE131928 (47), GSE163120 (21, 48), GSE89567 (49). GSE163120 can also be acquired by GBmap (https://doi.org/10.5281/zenodo.6962901). Group3 is available at DATE from https://registry.opendata.aws/cptac-3. Group6 is available at the Broad Institute Single-Cell Portal (https://singlecell.broadinstitute.org/single_cell/study/SCP503) (50). Two scRNA-seq datasets for normal brain tissue are available at Gene Expression Omnibus via GSE numbers: GSE126836 (51) and GSE140231 (52).

## Ethics statement

The studies involving human participants were reviewed and approved by the Scientific Ethics Committee at the First Affiliated Hospital of Xiamen University. The ethics committee waived the requirement of written informed consent for participation.

## Author contributions

SC, FZ, WM, ZW, and SW designed experiments, performed data analyses, and wrote the manuscript. SW, FH, XL, QC, SG, YY, YX, NX, and SC performed experiments and data visualization. SW and XK performed method comparison and assessment. FZ supervised the project. All authors contributed to the article and approved the submitted version.
