# Supplementary material for: Integrative analysis of single-cell transcriptomics reveals age-associated immune landscape of glioblastoma
Source: Front Immunol. 2023 Jan 24;14:1028775. doi: 10.3389/fimmu.2023.1028775 (PMC9903136; doi:10.3389/fimmu.2023.1028775)
Supplement: Supplementary file 1 [file DataSheet_1.pdf]

Supplementary Material

1 Supplementary Figures and Tables

1.1 Supplementary Figures

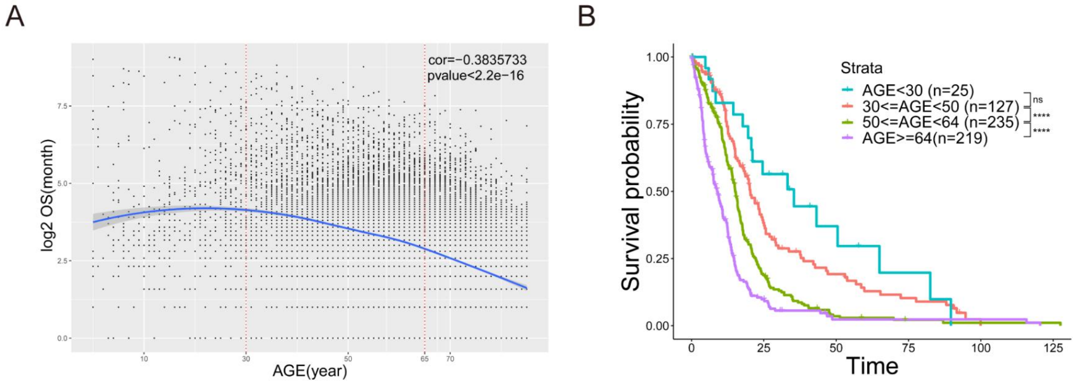

Supplementary Figure 1. Age is an independent risk factor of prognosis and overall survival. **(A)** Scatter plot of OS (logarithmically transformed) correlated with age from 20749 samples in SEER database. **(B)** Kaplan-meier curves of survival analysis used to compare the OS of GBM patients in TCGA database, divided into four groups according to age. ****(P < 0.0001), ns (no significance)

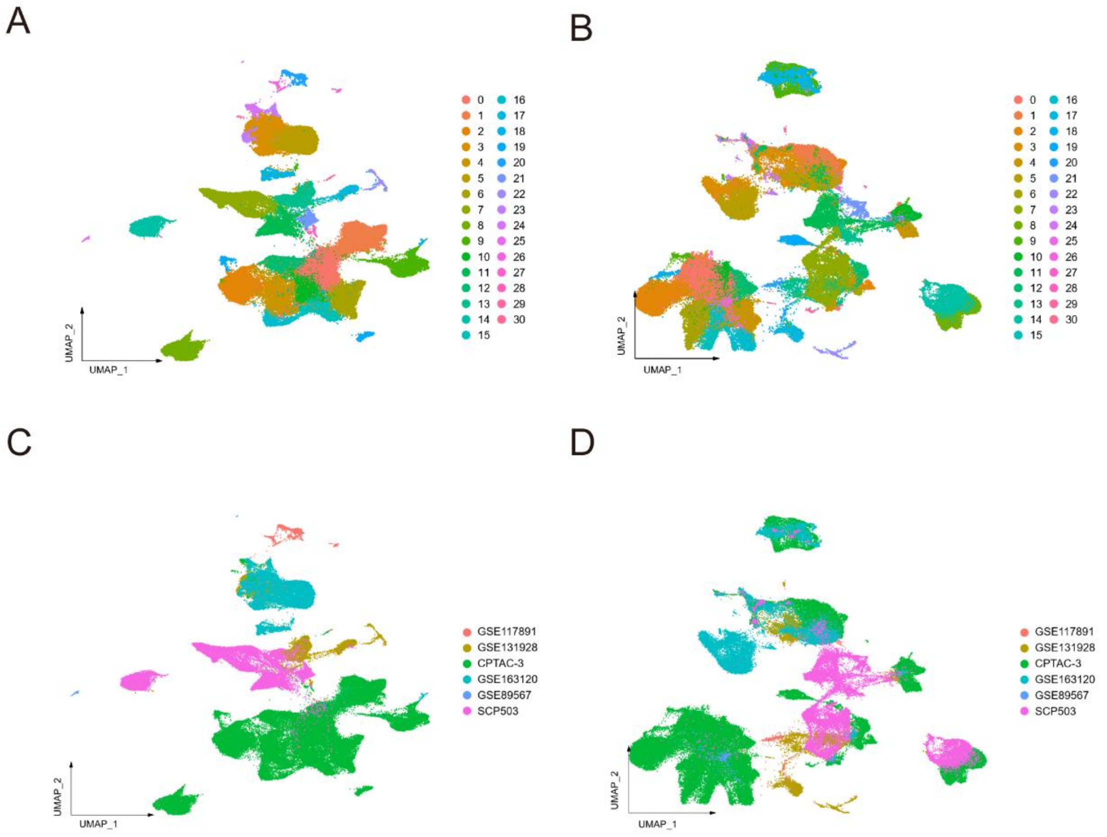

Supplementary Figure 2. Different methods to integrate scRNA-seq data. **(A-B)** UMAP plots colored by the cell type using seurat standard process (**A**) and harmony algorithm (**B**) in GBM. **(C-D)** UMAP plots colored by the scRNA-seq source using Seurat (**C**) and harmony (**D**) in GBM.

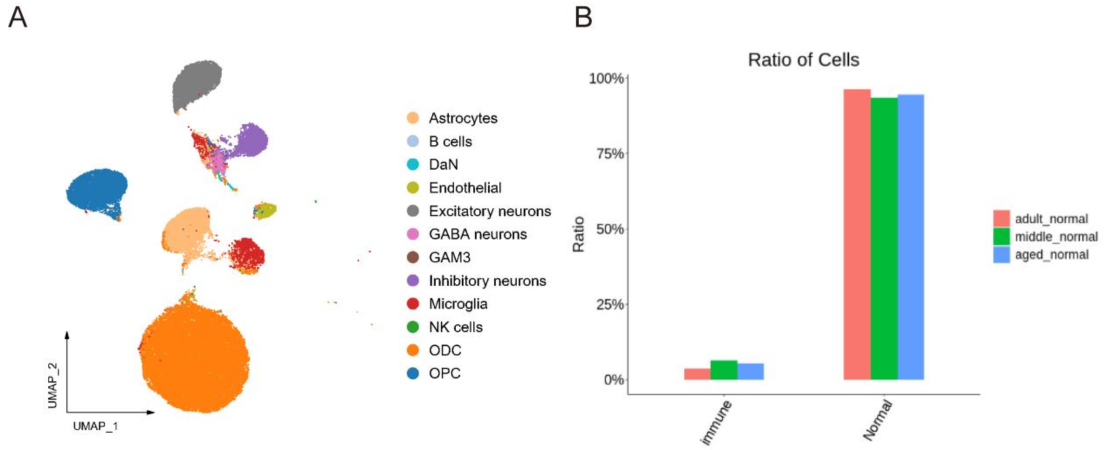

Supplementary Figure 3. Cell distribution of normal brain tissue. **(A)** UMAP plot of cells in normal human brain. **(B)** Histogram showing proprotion of immune cell or brain somatic cell in integrated normal brain scRNA-seq data, grouped by adult (n=2), middle aged(n=10) and aged (n=7).

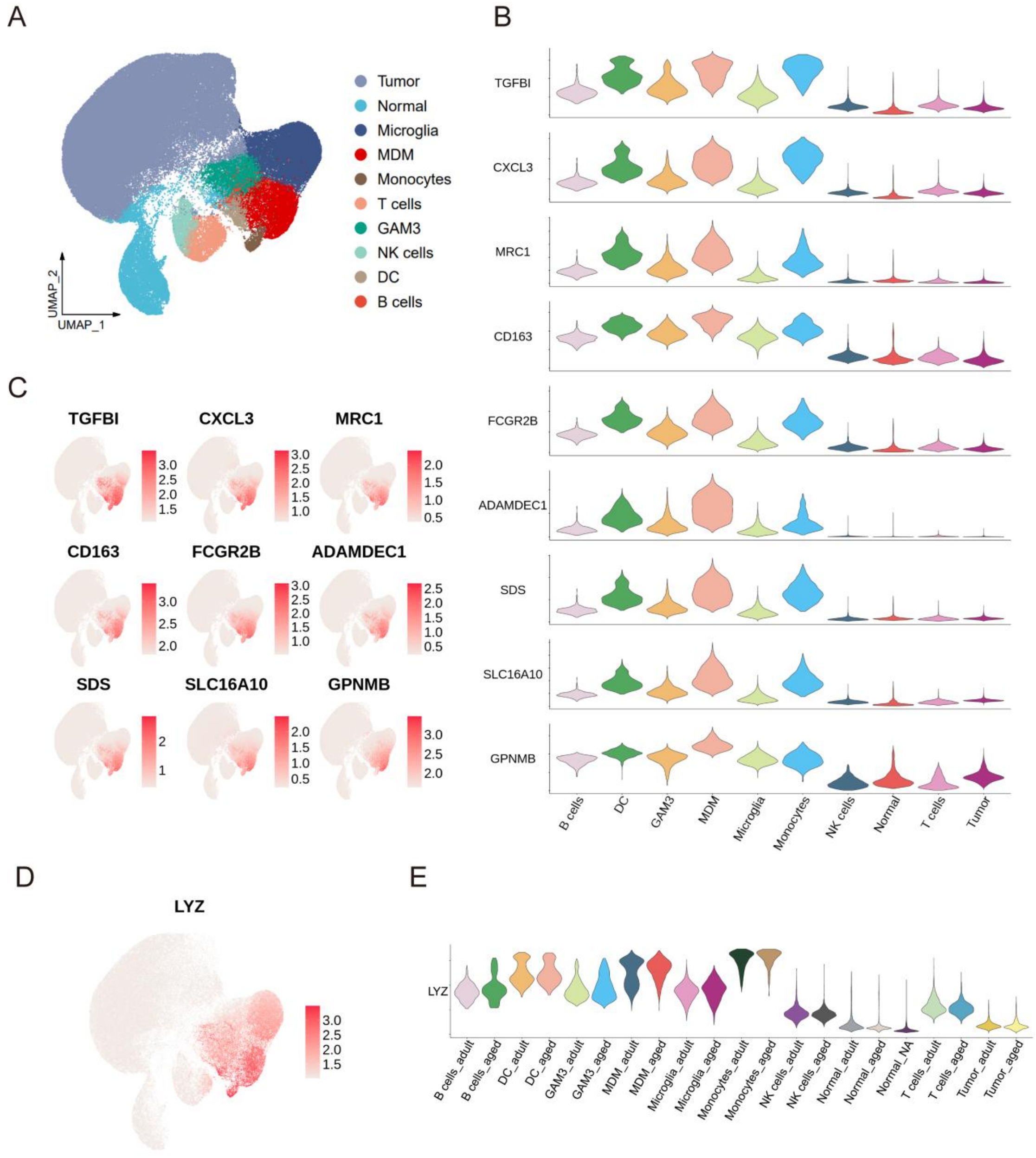

Supplementary Figure 4. Distribution and expression of MDM marker genes. **(A)** UMAP plot indicating distribution of individual cell types. **(B)** Violin plots showing top 9 MDM marker genes distribution in UMAP plot. **(C)** Feature plot showing top 9 MDM marker genes expression in each cell type. **(D)** Feature plot showing *LYZ* distribution in UMAP plot. **(E)** Violin plot showing *LYZ* expression in each cell type.

A

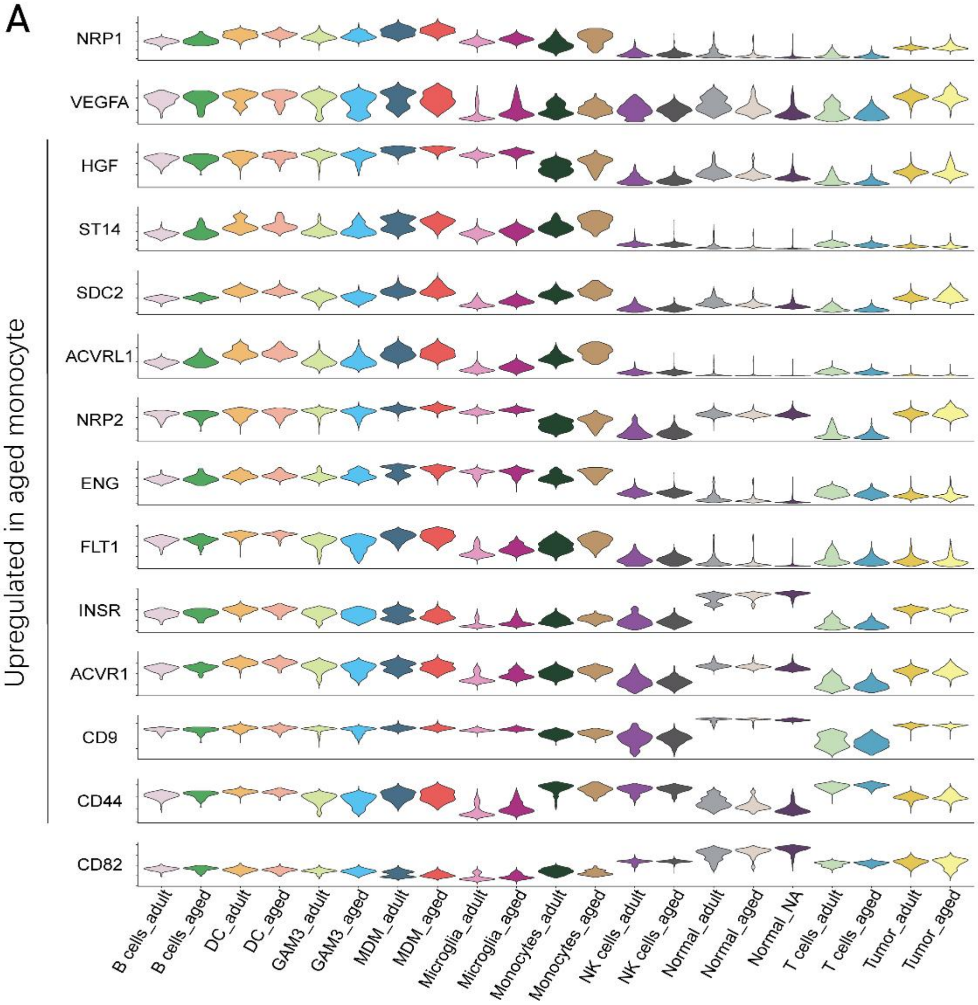

B

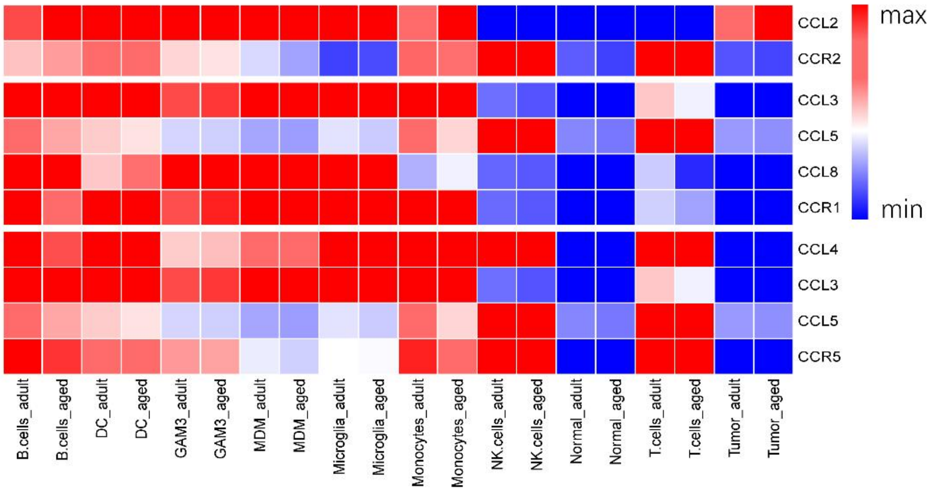

Supplementary Figure 5. Increased proliferation of monocytes, related to Figure 6A. **(A)** Violin plots showing the expression of monocyte receptors in each immune cell type, divided by adult and aged. **(B)** Heatmap of the expression of chemokines and receptors associated with monocyte recruitment.

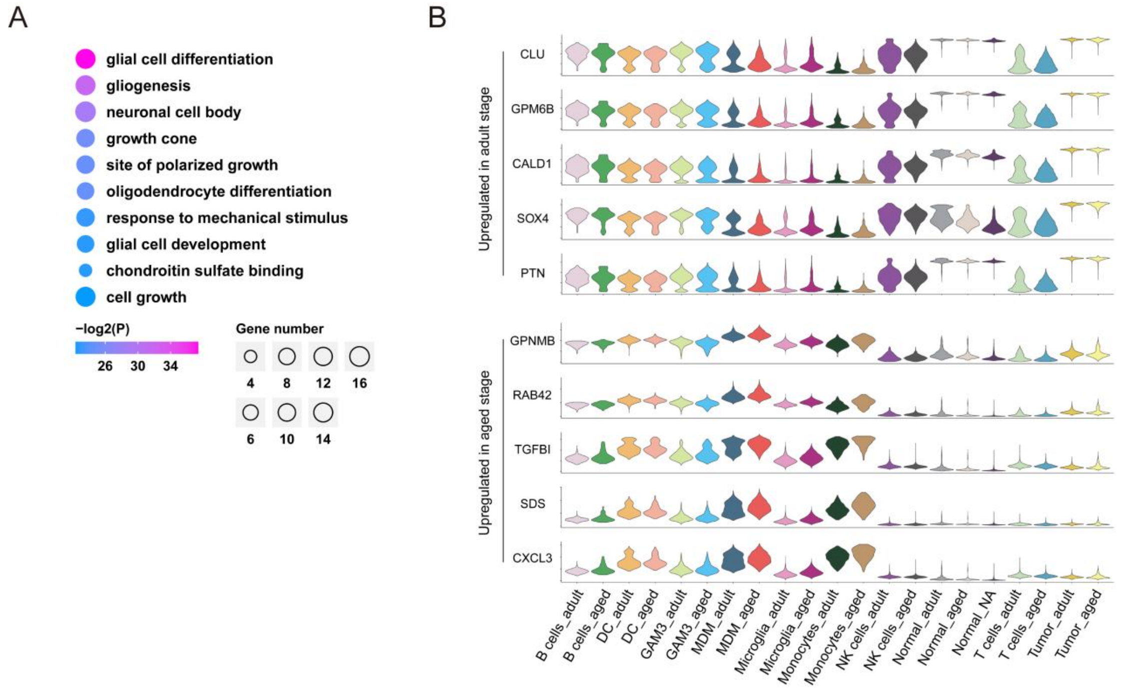

Supplementary Figure 6. Expression of differential genes between adult and aged MDMs. **(A)** GO (Gene ontology) analysis on top 100 relatively highly expressed genes in adult MDMs. Data displays top 10 enriched GO terms ranked by p values. Color indicates p values for GO term enrichment and circle size indicates the number of enriched genes for each GO term. **(B)** Violin plot showing the expression of genes in each immune cell types, which are up-regulated in adult or aged MDMs, divided by adult and aged.

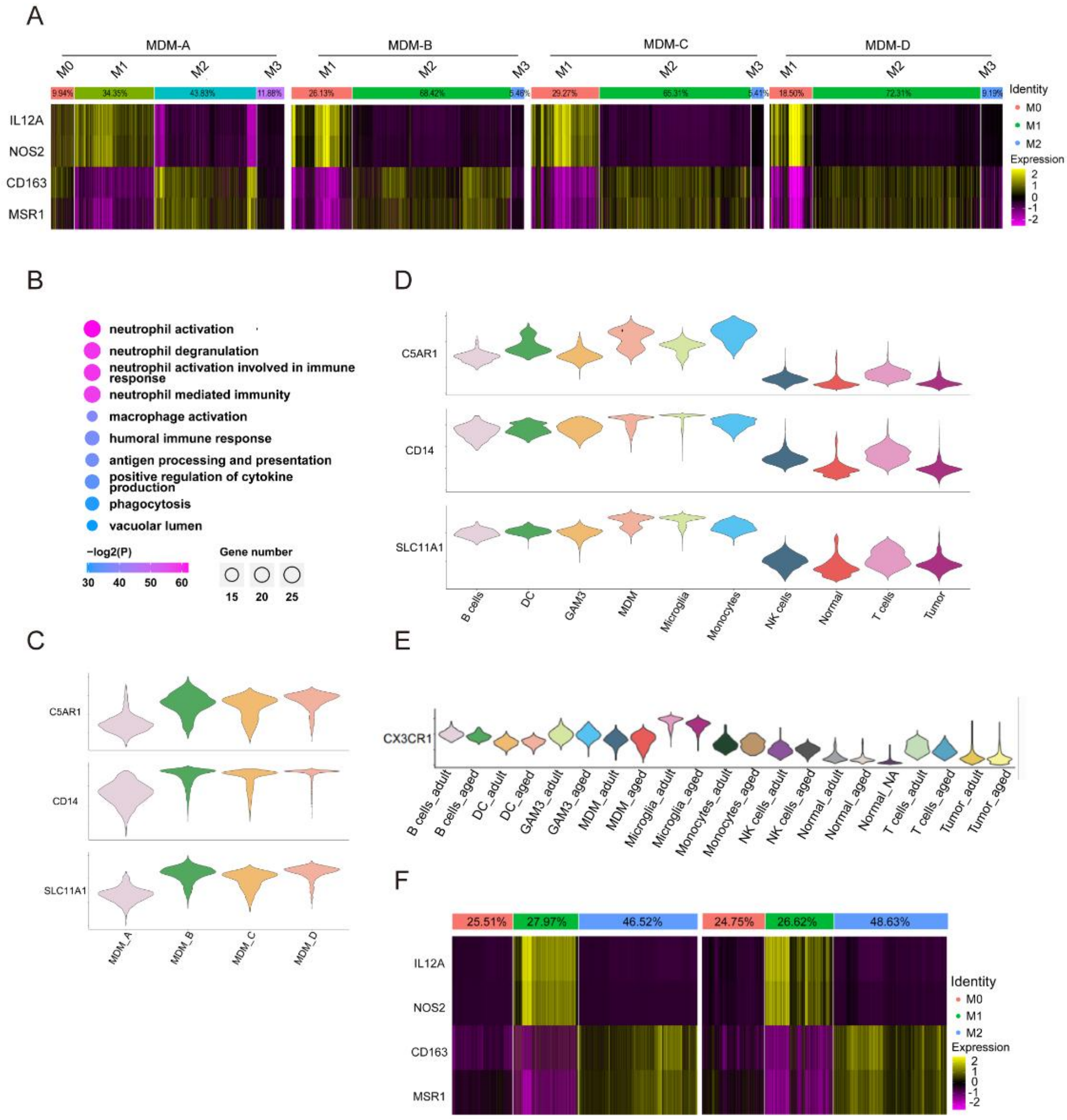

Supplementary Figure 7. The MDM-B subgroup but not microglia was the key to age-related OS reduction, related to Figure 5. **(A)** Heatmap showing the expression characteristics of the identified M1 and M2 subtypes cell marker genes in the MDM subgroups, and the ratio of each subtype is counted on the top row of columns. **(B)** GO (Gene ontology) analysis on top 100 relatively highly expressed genes in MDM-B across each MDM subgroup. Data display top 10 enriched GO terms ranked by p values. Color indicates p values for GO term enrichment and circle size indicates the number of enriched genes for each GO term. **(C)** Violin plot showing the expression of genes in each MDM subtypes, which up regulated in MDM-B subtype. **(D)** Violin plot showing the expression of genes in each immune cell type, which are up-regulated in MDM-B subtype. **(E)** Violin plot showing the expression of *CX3CR1* (marker of microglia) in each type of immune cells, divided into adult and aged cells. **(F)** Heatmap showing the expression characteristics of the identified M1 and M2 subtypes cell marker genes in the adult group (left) and the aged group (right), and the ratio of each subtype in adult or aged were counted on the top row of columns.

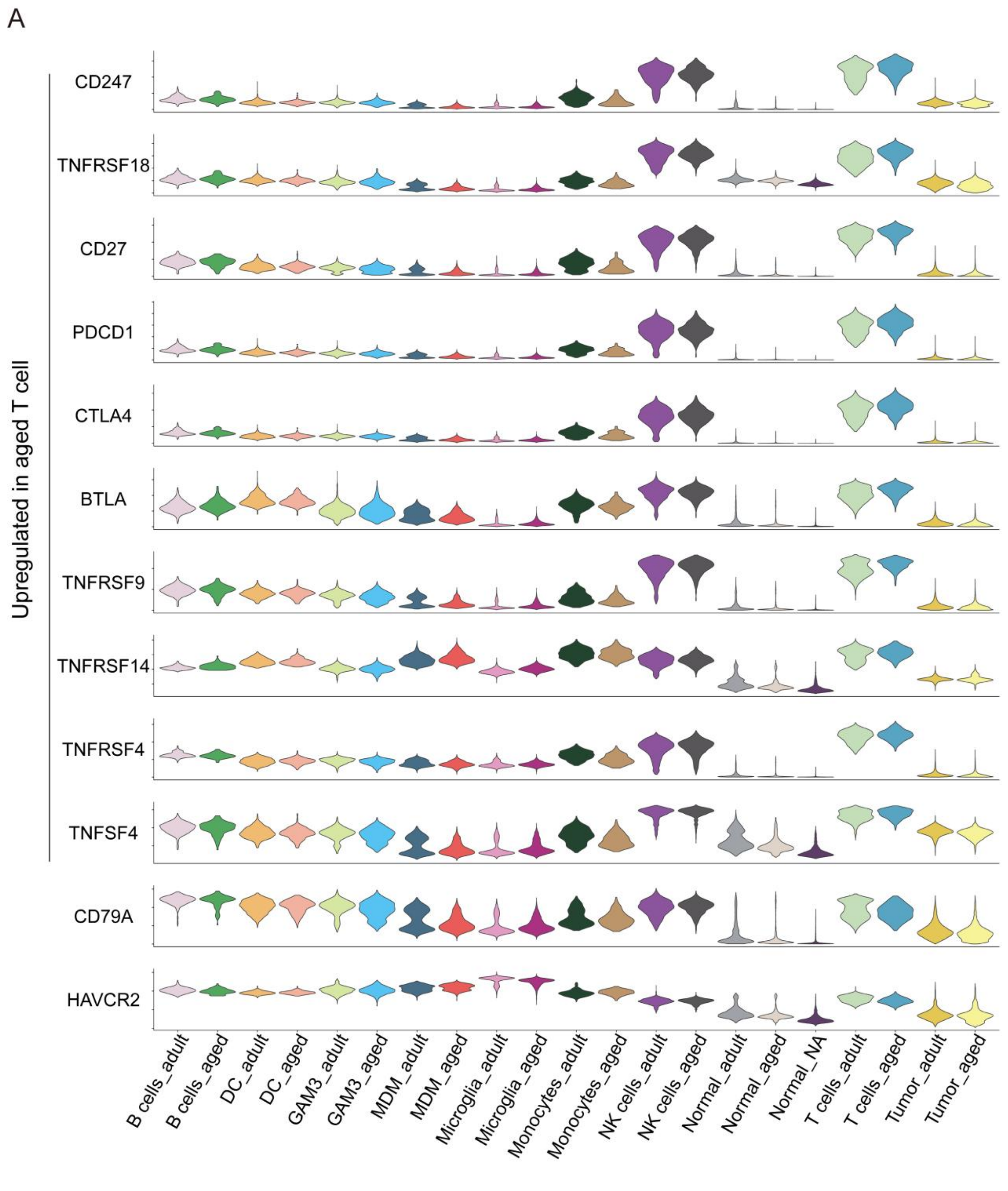

Supplementary Figure 8. MDMs regulate age-related OS by affecting T cells, related to Figure 7A. **(A)** Violin plot showing the expression of immune checkpoint genes in each immune cell type, divided by adult and aged.

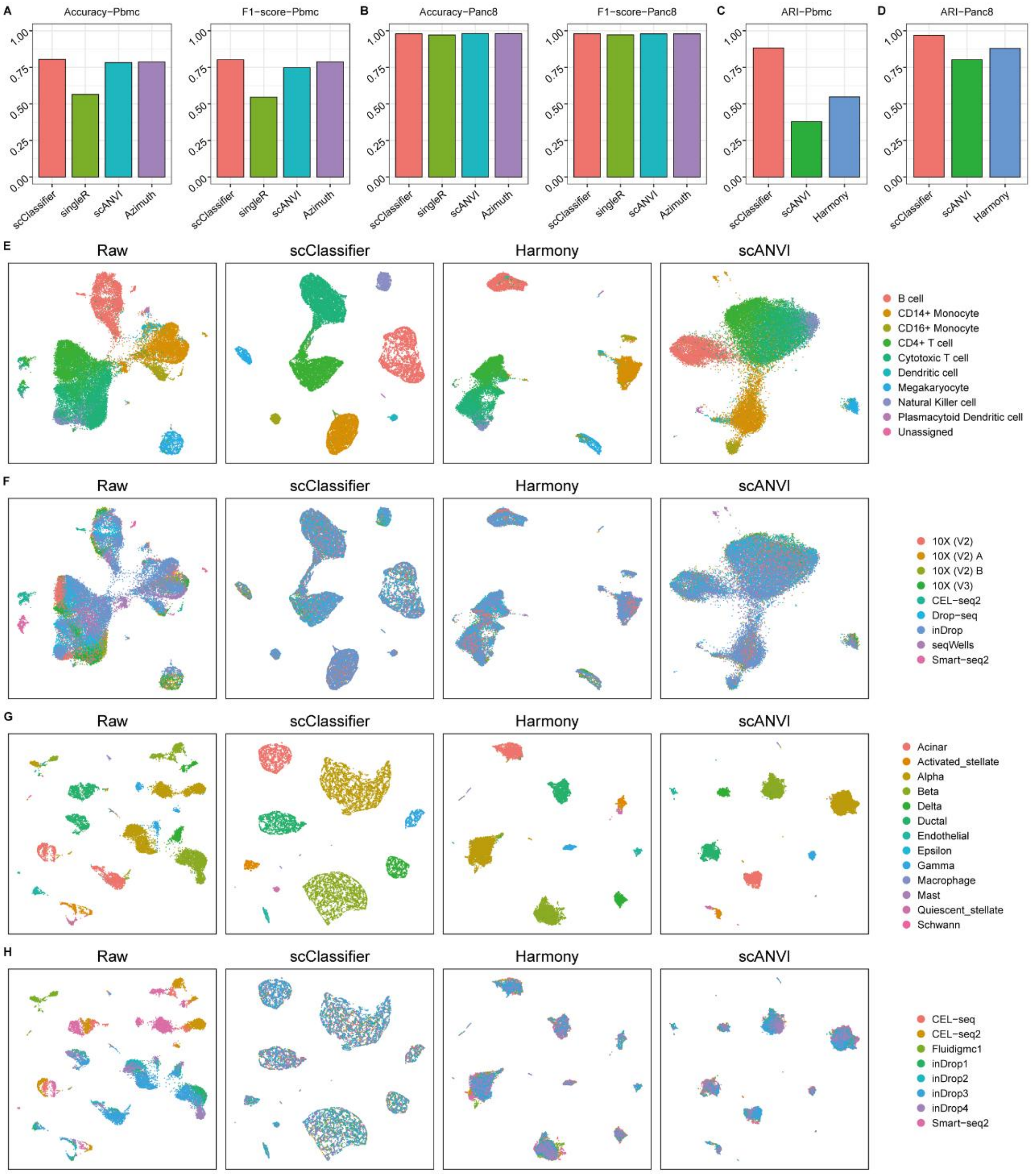

Supplementary Figure 9. The performance of our method with the standard cell-type annotation and integration methods. **(A-B)** Histograms showing the accuracy and F1 scores of singleR, scANVI, Azimuth and our method on PBMC **(A)** and panc8 **(B)** datasets. **(C-D)** Histograms showing the ARI scores of harmony, scANVI and our method on PBMC **(C)** and panc8 **(D)** datasets. **(E, G)** UMAP plots colored by the predicted cell-type using seurat standard process, harmony, scANVI and our method on PBMC **(E)** and panc8 **(G)** datasets. **(F, H)** UMAP plots colored by batch using seurat standard process, harmony, scANVI and our method on PBMC **(F)** and panc8 **(H)** datasets.

1.2 Supplementary Tables

Group	Total Cell Counts	Cell Counts		Number of Patients		Cell Type
		Adult	Aged	Adults	Aged	
1	1901	1645	256	3	1	tumor,immune
2	11786	0	11786	0	6	tumor,immune
3	84969	71053	13916	5	2	tumor,immune
4	15935	12259	3676	1	4	immune
5	415	415	0	1	0	tumor,immune
6	17982	N	N	N	N	tumor
Total	132988	85372	29634	10	13	

Supplementary Table 1. Statistical chart shows details of all samples that we collected, such as cell counts and number of patients.
